# The role of spontaneous effort during mechanical ventilation: normal lung versus injured lung

**DOI:** 10.1186/s40560-015-0083-6

**Published:** 2015-06-17

**Authors:** Takeshi Yoshida, Akinori Uchiyama, Yuji Fujino

**Affiliations:** Intensive Care Unit, Osaka University Hospital, 2-15 Yamadaoka, Suita, Osaka 565-0871 Japan; Department of Anesthesiology and Intensive Care Medicine, Osaka University Graduate School of Medicine, Suita, Japan

**Keywords:** Spontaneous breathing, Muscle paralysis, Lung injury, Pleural pressure, Transpulmonary pressure, ARDS, Pendelluft

## Abstract

The role of preserving spontaneous effort during mechanical ventilation and its interaction with mechanical ventilation have been actively investigated for several decades. Inspiratory muscle activities can lower the pleural components surrounding the lung, leading to an increase in transpulmonary pressure when spontaneous breathing effort is preserved during mechanical ventilation. Thus, increased transpulmonary pressure provides various benefits for gas exchange, ventilation pattern, and lung aeration. However, it is important to note that these beneficial effects of preserved spontaneous effort have been demonstrated only when spontaneous effort is modest and lung injury is less severe. Recent studies have revealed the ‘dark side’ of spontaneous effort during mechanical ventilation, especially in severe lung injury. The ‘dark side’ refers to uncontrollable transpulmonary pressure due to combined high inspiratory pressure with excessive spontaneous effort and the injurious lung inflation pattern of Pendelluft (i.e., the translocation of air from nondependent lung regions to dependent lung regions). Thus, during the early stages of severe ARDS, the strict control of transpulmonary pressure and prevention of Pendelluft should be achieved with the short-term use of muscle paralysis. When there is preserved spontaneous effort in ARDS, spontaneous effort should be maintained at a modest level, as the transpulmonary pressure and the effect size of Pendelluft depend on the intensity of the spontaneous effort.

## Introduction

The role of spontaneous breathing during mechanical ventilation has been discussed for several decades [1-4]. From a physiological point of view, spontaneous breathing during mechanical ventilation provides various beneficial effects, including the maintenance of the end-expiratory lung volume, predominant dorsal ventilation, better gas exchange, and prevention of diaphragmatic dysfunction [1-9]. Thus, spontaneous effort has traditionally been encouraged to be preserved during mechanical ventilation [1,2]. Recent experimental studies, however, have shed light on the negative impacts of spontaneous breathing, especially in severe forms of ARDS [10-12]. Further, recent clinical studies have revealed the beneficial impacts of eliminating all muscle activities by neuromuscular blocking agents in severe forms of ARDS [13-16]. These different impacts of spontaneous breathing during mechanical ventilation may be explained by different inflation patterns that are observed in normal (*fluid-like*) lungs versus injured (*solid-like*) lungs and transpulmonary pressure. The goals of this review are to summarize the physiological mechanisms of different lung ventilation in normal lungs versus injured lungs, raise important concerns about spontaneous breathing in ARDS, and present an updated discussion on the role of spontaneous breathing and muscle paralysis during mechanical ventilation in ARDS.

## Review

### Mechanism to determine the diaphragmatic force

As the main inspiratory muscle, the diaphragm contributes 72% of tidal breath, and its role in respiratory mechanics and gas exchange is also very significant [17]. When spontaneous effort starts, diaphragmatic fibers develop tension and shorten. As a result, the dome of the diaphragm, which essentially corresponds to the central tendon, descends relative to the costal insertions of the muscle, resulting in two main effects [18]. *First*, it expands the thoracic cavity along its craniocaudal axis. Accordingly, pleural pressure (*P*_pl_) falls and lung volume increases. *Second*, it produces a caudal displacement of the abdominal viscera and an increase in abdominal pressure, which, in turn, pushes the ventral abdominal wall outward [18]. This pressure-generating capacity of the diaphragm is traditionally accepted to be determined by several factors, but the force-length relationship of the diaphragm and its radius of curvature are the most significant.

#### The force-length relationship of the diaphragm

In dogs, cats, rabbits, and humans, the negative swings in *P*_pl_ with phrenic nerve stimulation have been proven to decrease with increasing end-expiratory lung volume before starting phrenic nerve stimulation [19-22]. As a typical example, Pengelly et al. reported that in cats, the negative swings in *P*_pl_ with phrenic nerve stimulation decreased rapidly and continuously from −13 cm H_2_O to −8.5 (or −10.5) cm H_2_O when inflated with a volume of 20 (or 10) ml from functional residual capacity [22]. Thus, the pressure-generating capacity of the diaphragm decreases when the end-expiratory lung volume increases. This observation is explained by the mechanism known as the force-length relationship of the diaphragm, which is the idea that the isometric force developed by a muscle decreases when its length decreases [19,22-24]. As the length of the muscle bundle increases, the active force gradually increases until a maximum is reached, and it then decreases again. The length corresponding to the maximum active force is usually referred to as the optimal length and is typically achieved at functional respiratory capacity [18]. When the lung volume in animals and humans is increased from residual volume to total lung capacity, the diaphragmatic fibers shorten by 30–40%.

#### The radius of curvature of the diaphragm

The diaphragm is a curved surface, so the pressure difference across it is proportional to the muscle tension and inversely proportional to the radius of curvature of the muscle (Laplace’s law). As the shape of the diaphragm becomes flatter, the mechanical advantage of converting force into pressure diminishes [20]. Thus, the pressure-generating capacity of the diaphragm is *theoretically* diminished by increasing the radius of its curvature [20,22,23]. However, in humans, as well as in dogs, the radius of the diaphragm curvature during spontaneous effort remains mostly constant or changes little, independent of the end-expiratory lung volume [25]. At an extreme condition (i.e., phrenic nerve stimulation), the radius of the diaphragm curvature increases sharply [25]. Thus, the pressure-generating capacity of the diaphragm is primarily determined by its force-length relationship, and the shape of the diaphragm is only important during extreme muscle shortening [23].

### Interaction of inspiratory muscles and mechanical ventilation

From a physiological point of view, the inflation of the lung occurs when the pressure on the lung surface (i.e., *P*_pl_) becomes sufficiently negative due to inspiratory muscle contractions or when pressure in the airway (from positive-pressure ventilation) becomes sufficiently positive. In muscle paralysis (i.e., without inspiratory muscles effort), airway pressure applied from positive-pressure ventilation is consumed to inflate not only the lung but also the chest wall (rib cage + abdomen). Thus, the portion of the applied pressure inflating the lung (transpulmonary pressure) could vary widely, depending on the chest wall characteristics [26,27].

$$ \mathrm{Transpulmonary}\kern0.24em \mathrm{pressure}\;\left({P}_{\mathrm{L}}\right)=\mathrm{Airway}\kern0.24em \mathrm{pressure}\;\left({P}_{\mathrm{aw}}\right)\hbox{--} \mathrm{Pleural}\kern0.24em \mathrm{pressure}\kern0.24em \left({P}_{\mathrm{pl}}\right) $$

where transpulmonary pressure is the pressure needed to inflate the lung, airway pressure is the pressure applied by positive-pressure ventilation via the trachea, and pleural pressure is the lung surface pressure imposed by the chest wall.

**Figure 1:**
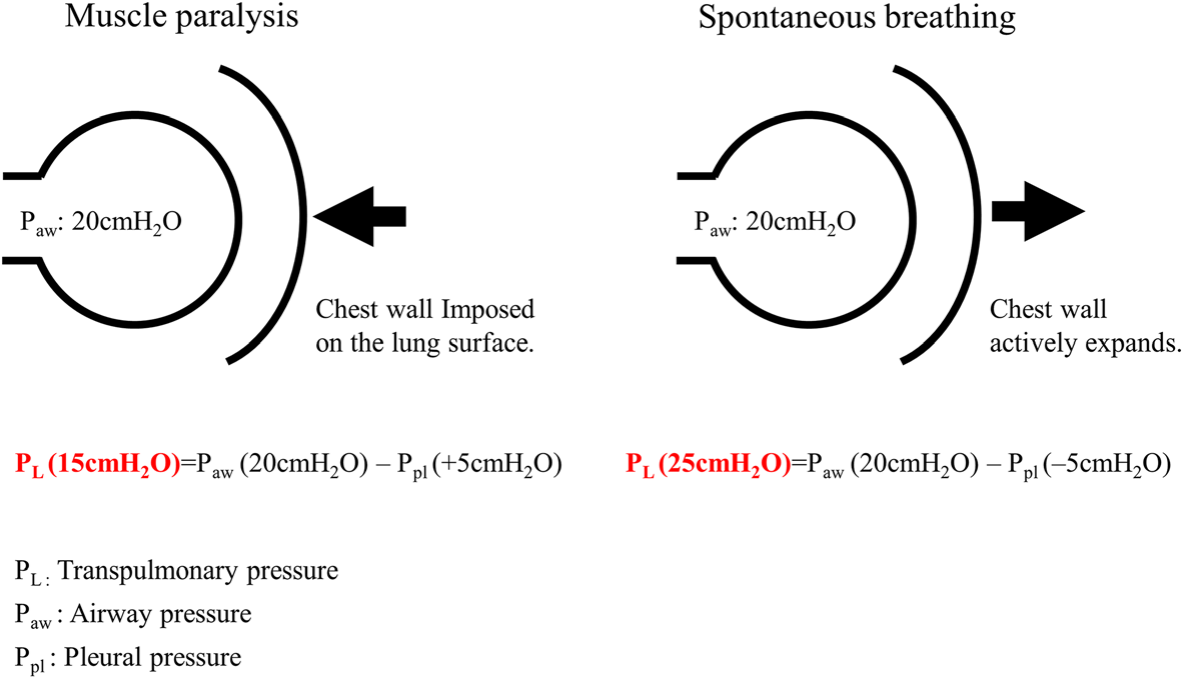
**Transpulmonary pressure difference: muscle paralysis vs. spontaneous breathing.** Diaphragmatic contraction can elevate transpulmonary pressure with the same airway pressure applied in muscle paralysis, by altering the pleural components surrounding the lung.

For instance, when we deliver 20 cm H_2_O of inspiratory airway pressure by the mechanical ventilator, part of *P*_aw_ is consumed to inflate the chest wall unless spontaneous effort is preserved. As a result, *P*_L_ to inflate the lung is 15 cm H_2_O (Figure 1).

**Figure 2:**
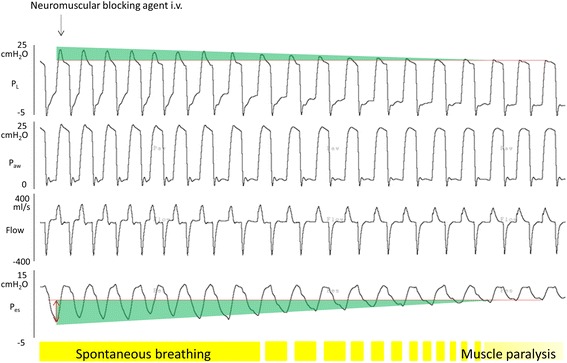
**Transition phase from spontaneous breathing to muscle paralysis in a rabbit.** A lung-injured animal was ventilated with assisted pressure control mode. We recorded continuously waveforms of transpulmonary pressure, airway pressure, flow, and esophageal pressure without any change in ventilatory settings, after injection of neuromuscular blocking agent. When spontaneous breathing during mechanical ventilation is diminished, the negative swing in esophageal pressure is decreasing. As a result, inspiratory transpulmonary pressure decreases. Note that inspiratory transpulmonary pressure linearly correlates with the intensity of spontaneous breathing effort.

On the other hand, inspiratory muscle contraction can elevate *P*_L_ with the same *P*_aw_ applied in muscle paralysis (Figures 1 and 2). During mechanical ventilation, these two different types of pressure work to inflate the lung together. Thus, when spontaneous breathing is preserved during positive-pressure ventilation, negative changes in *P*_pl_ may be coupled with positive pressure changes from the ventilator, magnifying *P*_L_. For instance, when we deliver 20 cm H_2_O of inspiratory airway pressure by the mechanical ventilator, a negative change in *P*_pl_ is generated by inspiratory muscle contractions (for instance, −5 cm H_2_O) and is continued until the inspiratory airway pressure reaches its peak. As a result, *P*_L_ is 25 cm H_2_O (Figure 1). These inspiratory muscle activities can lower the pleural components surrounding the lung, leading to an increase in *P*_L_ when spontaneous breathing effort is preserved during mechanical ventilation [28]. Thus, spontaneous breathing is traditionally encouraged during mechanical ventilation [1,2] because it is thought to provide lung expansion at lower levels of *P*_aw_, which is a strategy that would result in better local (especially dependent) lung aeration, thereby enhancing gas exchange and potentially improving hemodynamics [2,5-7].

### Ventilation pattern with preserved spontaneous effort in a normal lung

**Figure 3:**
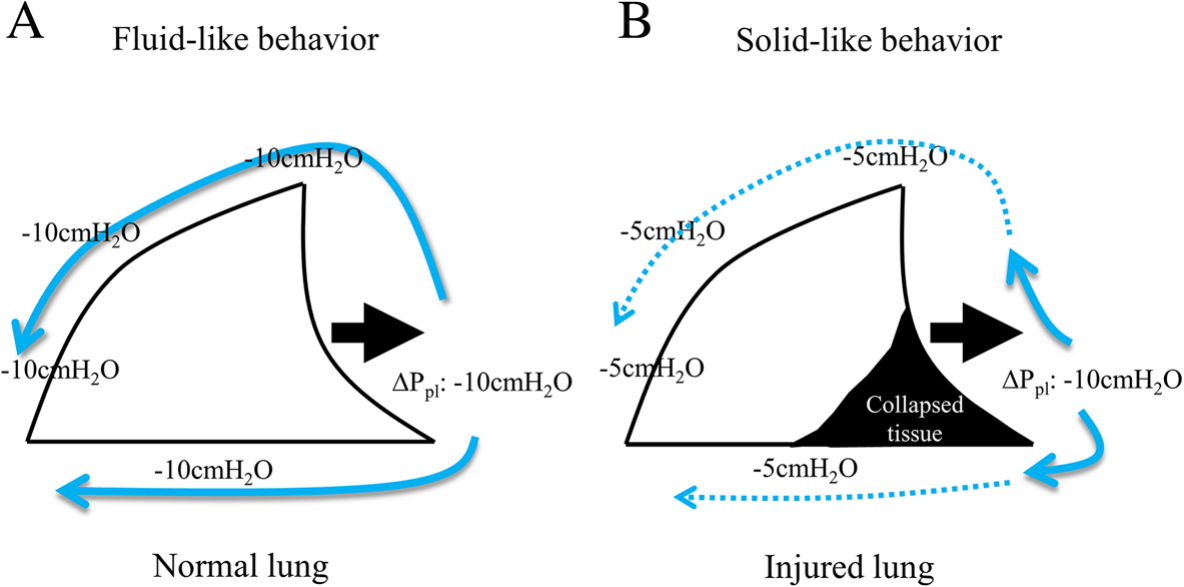
**Fluid-like behavior presented in normal lung vs. solid-like behavior presented in injured lung. (A)** The normal lung is traditionally considered to be a continuous elastic system—exhibiting fluid-like behavior—such that distending pressure applied to a local region of the pleura (the negative swing in pleural pressure generated by diaphragmatic contraction is −10 cm H_2_O) becomes generalized over the whole lung (pleural) surface (the negative swings in pleural pressure at any regions are the same −10 cm H_2_O). **(B)** In injured lung, the negative swing in pleural pressure generated by diaphragmatic contraction is not uniformly transmitted, but rather concentrated in the dependent lung regions, thus a huge difference in negative pleural pressure between nondependent and dependent lung regions was generated at the early phase of inspiration, causing Pendelluft. Adapted with permission of the Wolters Kluwer Health (Ref. [36]).

Classical physiological studies have shown that pressures applied to the lung surface (through the contraction of inspiratory muscles) or to the airways (through positive-pressure ventilation) re-equilibrate by a special rearrangement of the forces within the lung so that the lung is considered to behave as a continuous elastic system, presenting with a fluid-like behavior [29,30] (Figure 3). This means that local swings in *P*_pl_, as during usual muscle contraction, tend to be transmitted all over the lung surface, creating a fairly uniform increase in *P*_L_ [31-35]. This is one of the justifications for using the esophageal pressure (*P*_es_) to estimate overall fluctuations in *P*_pl_ in normal subjects. It is important to note that the uniform distribution of forces presented in a normal lung is the basis of an occlusion test to adjust the appropriate position of the esophageal balloon. The relationship between the change in *P*_pl_ and the change in *P*_aw_ should not present near unity without a uniform distribution of forces during an occlusion test. Agostoni put the cylinder on the pleural surfaces from the apex to base, even on the diaphragmatic surface, and as a result, the local changes in *P*_pl_ during spontaneous effort were not systematically different among the pleural regions [31,32]. For this uniform distribution of forces, the preservation of spontaneous breathing effort achieves a uniform increase in ventilation at a relatively low airway pressure in normal situations (i.e., normal lung and normal respiratory drive).

### Ventilation pattern with preserved spontaneous effort in injured lungs

**Figure 4:**
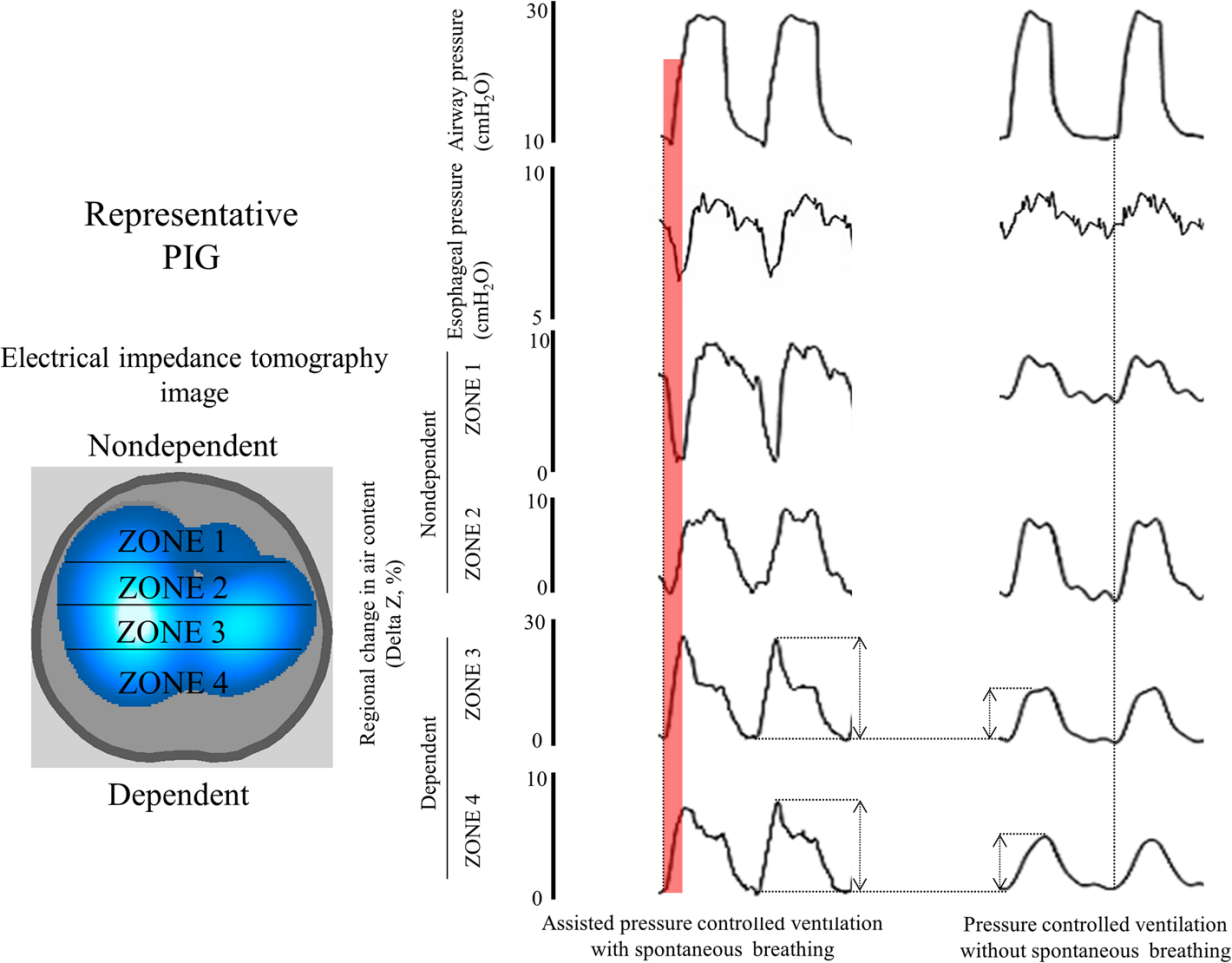
**EIT waveforms in experimental lung injury—spontaneous versus mechanical breaths.** Note that the early inflation in the dependent region (*Zones 3 and 4*) was accompanied by concomitant deflation of nondependent region (*Zones 1 and 2*), indicating movement of air from nondependent to dependent lung (i.e. Pendelluft). Note that under the same tidal volume, spontaneous breathing during mechanical ventilation unsuspectedly increased dependent lung inflation (*Zones 3 and 4*) due to Pendelluft. Adapted with permission of the American Thoracic Society Copyright © 2014 American Thoracic Society (Ref. [12]).

In contrast to the fluid-like behavior that is observed in normal lungs, the change in *P*_pl_ generated by inspiratory muscle contractions in injured lungs is not uniformly transmitted across the lung surface, but rather is concentrated in dependent lung regions [12] (Figure 3). This locally elevated change in *P*_L_ causes unsuspected local overstretch in dependent lung regions, accompanying an alveolar air shift from nondependent (fluid-like, more recruited regions) to dependent (solid-like, less recruited regions) parts of the lung (i.e., Pendelluft) (Figure 4) [12].

It is important to note that the pattern of lung inflation is different in the presence of lung injury. In the injured lung, Pendelluft occurs as a result of the development of a more negative swing in *P*_pl_ in the dependent lung than in the nondependent lung. Atelectatic tissue may behave less like a fluid and more like a frame of ‘solid’ areas resisting to shape deformation. In this setting, part of the mechanical energy generated by the inspiratory muscle contractions would be exerted on local lung deformation rather than being transmitted to the rest of the lung, thus resulting in imperfect elastic anisotropic inflation [12].

Inspiratory *P*_L_ and the effect size of Pendelluft become larger as spontaneous effort increases in strength during mechanical ventilation (Figure 2 and ref. [12]). Thus, mild spontaneous effort may be beneficial to recruit the collapsed lung, while excessive spontaneous effort could cause local overstretch due to injuriously high *P*_L_ and the large effect size of Pendelluft [12,36].

### Controversial effects of spontaneous breathing in ARDS

#### The role of spontaneous breathing in mild-moderate ARDS

**Figure 5:**
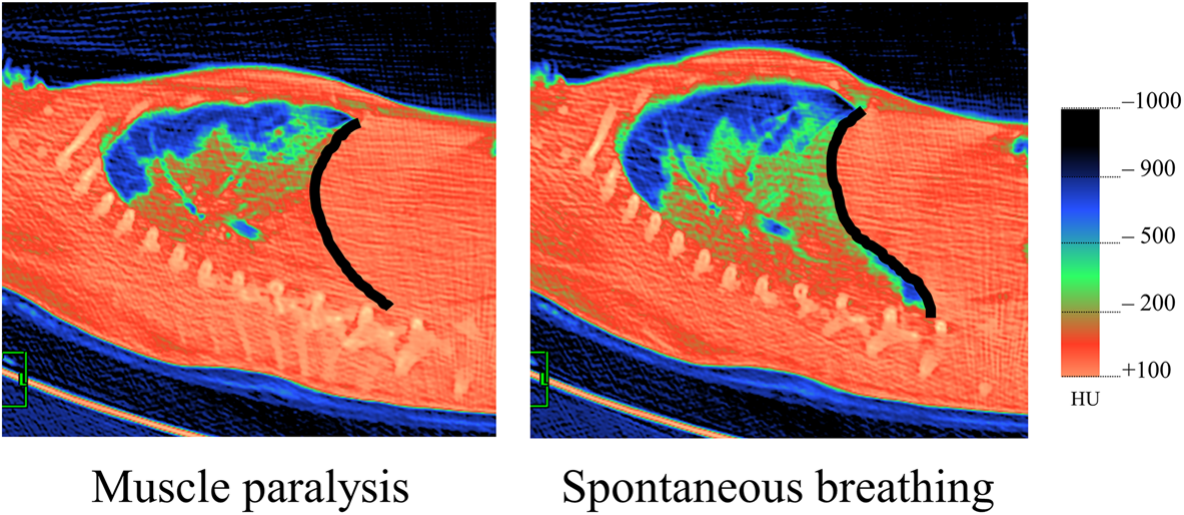
**CT images in experimental lung injury—muscle paralysis vs. spontaneous breathing.** Both dynamic CT images of the same anatomical, sagittal level at end-expiration are shown to compare the end-expiratory lung volume and the shape of the diaphragm. CT images are colored according to their Hounsfield units densities. The black lines indicate the diaphragm at end-expiration. These dynamic CT images were continuously taken after injection of neuromuscular blocking agent, without any change in ventilatory settings. Spontaneous breathing effort restored the end-expiratory lung volume due to diaphragmatic muscle tone. Once diaphragm was paralyzed, diaphragm shifted to cranial direction, resulting in large collapse in dorsal lung regions. Note that this happened because inadequate (low) PEEP was applied.

It is important to note that the evidence for beneficial effects of spontaneous breathing has been gathered in normal lungs and less severe forms of ARDS with modest ventilatory demands [2,3,5-7,11]. Spontaneous breathing effort during mechanical ventilation improves gas exchange and has been associated with better lung aeration in CT analysis in experimental and clinical studies with less severe forms of ARDS [2,5-7,11]. The plausible explanation for the beneficial effects of spontaneous effort is the alternation of the pleural compartment surrounding the lung. Gentle inspiratory muscle contractions expand the lung actively, leading to an increase and sustainment in *P*_L_ [11,28]. Continuous tonic activity of the diaphragm is effective for maintaining end-expiratory lung volume [37]. Paralysis shifts the diaphragm to the cranial direction and increases *P*_pl_, resulting in a significant decrease in the end-expiratory lung volume (Figure 5). A tidal increase in *P*_L_ during inspiration also achieves homogeneous ventilation. In 2001, Putensen et al. performed a randomized clinical study in trauma patients with acute lung injury (note that subjects were not ARDS) and found that the preserved spontaneous effort during mechanical ventilation improves oxygenation and shortens durations of ventilatory support and ICU stays compared with a muscle paralysis group [2]. Several issues for optimizing the beneficial effects of spontaneous effort during mechanical ventilation should be addressed. *First*, the plateau pressure that was applied in clinical (and experimental) studies that demonstrated the benefits of spontaneous breathing could be kept relatively low because the lung injury was less severe [2,5-7,11,38]. The review of biphasic positive airway pressure (BIPAP) ventilation (ventilatory mode to facilitate spontaneous breathing effort) performed during the past 24 years demonstrates that plateau pressures applied during BIPAP ventilation is less than 20 cm H_2_O in patients with ARDS [39]. In contrast, plateau pressures applied in clinical studies showing the beneficial effects of muscle paralysis on severe ARDS were higher (25–27.5 cm H_2_O), reflecting the severity of ARDS [13-15]. *Second,* spontaneous effort is generally modest in less severe forms of ARDS, which is evident from the lesser duration and lower amplitude of the negative swings in *P*_pl_ that diaphragmatic contraction generates [11].

Thus, it is important to emphasize that it is necessary to avoid strong spontaneous efforts (i.e., not high ∆pleural pressure) and maintain relatively low plateau pressure (i.e., not high ∆airway pressure) in order to prevent the large effect of Pendelluft and injuriously high *P*_L_.

#### The role of spontaneous breathing in severe ARDS

In severe ARDS, however, spontaneous effort during mechanical ventilation is difficult to control and becomes unfavorable [11]. Several plausible explanations can be offered. *First*, the increases in *P*_L_ are expected to be greatest in more severe ARDS because in such cases, higher plateau pressure is required from the ventilator, reflecting an impaired respiratory system compliance. In addition, greater diaphragmatic force is often generated by the patient, reflecting the high levels of dyspnea [11]. Injuriously high *P*_L_ proved to worsen histological lung injury in our previous animal studies [10,11]. *Second*, we recently revealed an injurious ventilation pattern caused by strong spontaneous effort, i.e., Pendelluft, which is the displacement of gas from nondependent (more recruited) lung to dependent (less recruited) lung during early inspiration [12]. Despite the limitation of tidal volume to less than 6 mL/kg, strong diaphragmatic contraction resulted in unsuspected local overstretch of the dependent lung due to the large effect of Pendelluft, leading to tidal recruitment in dynamic CT acquisitions. Matching this degree of regional overstretch during neuromuscular paralysis required an overall tidal volume of 15 mL/kg (i.e., *a highly injurious lung stretch*) [12]. Importantly, this injurious ventilation pattern cannot be suspected by using conventional monitoring, such as airway pressure monitoring, flow monitoring, and even esophageal pressure monitoring. Thus, a lung-protective ventilation strategy (i.e., the limitation of plateau pressure and tidal volume) is not effective for reducing the risk of ventilator-induced lung injury unless spontaneous breathing effort during mechanical ventilation is carefully controlled at a modest level.

We often find that the demands of spontaneous breathing effort in severe ARDS is much higher than in less severe ARDS, and as a result, it is quite difficult to control the intensity of spontaneous effort by sedatives [11]. This is likely due to metabolic/respiratory acidosis, hypercapnia, or decreased end-expiratory lung volume due to a large amount of collapsed tissues (mentioned above). So far, the most effective, established strategy is to eliminate spontaneous effort completely by the initiation of neuromuscular blocking agents [13-15]. In the ACURASYS study, the placebo group (i.e., no neuromuscular blockade use) had a higher incidence of barotrauma, even at the comparable plateau pressure and tidal volume, to the muscle paralysis group [15], suggesting spontaneous effort may have generated injuriously high *P*_L_ and unsuspected local overstretch of dependent lung regions, which is associated with Pendelluft. However, another simple, safe strategy to reduce the intensity of spontaneous effort needs to be promptly established. As indicated above, the negative swings in *P*_pl_ generated by diaphragmatic contraction is proven to decrease linearly with increasing end-expiratory lung volume and radius of curvature [19-22]. Considering the mechanical property of the diaphragm to generate the pressure, optimized PEEP with lung recruitment might be effective for reducing the intensity of spontaneous effort by restoring the end-expiratory lung volume and reducing the diaphragmatic radius of curvature. Indeed, previous studies support this aspect because spontaneous breathing effort is typically weaker on high PEEP level than that on low PEEP level during BIPAP [40,41]. This aspect should be explored in future studies.

## Conclusion

It is important to balance muscle paralysis versus spontaneous breathing during mechanical ventilation in ARDS, depending on the severity of ARDS, the timing of ARDS, and the ventilatory demands. In the early stage of severe ARDS, partial ventilatory support to promote spontaneous breathing should be avoided, and muscle paralysis may be effective to strictly control *P*_L_ within the safe range, thus preventing Pendelluft. In less severe forms of ARDS and after the short-term use of muscle paralysis in severe ARDS, spontaneous breathing should be facilitated using partial ventilatory support while avoiding strong spontaneous effort and high plateau pressure.
